# Next generation of anti-PD-L1 Atezolizumab with enhanced anti-tumor efficacy in vivo

**DOI:** 10.1038/s41598-021-85329-9

**Published:** 2021-03-11

**Authors:** Maohua Li, Rongqing Zhao, Jianxin Chen, Wenzhi Tian, Chenxi Xia, Xudong Liu, Yingzi Li, Song Li, Hunter Sun, Tong Shen, Wenlin Ren, Le Sun

**Affiliations:** 1 AbMax BioPharmaceuticals Co., LTD, 99 Kechuang 14th Street, BDA, Beijing, 101111 China; 2 AnyGo Technology Co., LTD, Beijing, China; 3 ZhenGe Biotechnology Co., LTD, Shanghai, China; 4 ImmuneOnco Biopharma (Shanghai) Co., LTD, Shanghai, China

**Keywords:** Molecular engineering, Protein design, Drug development

## Abstract

FDA-approved anti-PD-L1 antibody drug Atezolizumab is a human IgG1 without glycosylation by an N297A mutation. Aglycosylation of IgG1 has been used to completely remove the unwanted Fc-mediated functions such as antibody-dependent cytotoxicity (ADCC). However, aglycosylated Atezolizumab is unstable and easy to form aggregates. Here, we report the development of the anti-PD-L1 antibody drug Maxatezo, a glycosylated version of Atezolizumab, with no ADCC activity, better thermo-stability, and significantly improved anti-tumor activity in vivo. Using Atezolizumab as the starting template, we back-mutated A297N to re-install the glycosylation, and inserted a short, flexible amino acid sequence (GGGS) between G237 and G238 in the hinge region of the IgG1 heavy chain. Our data shows that insertion of GGGS, does not alter the anti-PD-L1′s affinity and inhibitory activity, while completely abolishing ADCC activity. Maxatezo has a similar glycosylation profile and expression level (up to 5.4 g/L) as any normal human IgG1. Most importantly, Maxatezo’s thermal stability is much better than Atezolizumab, as evidenced by dramatic increases of Tm1 from 63.55 °C to 71.01 °C and T_agg_ from 60.7 °C to 71.2 °C. Furthermore, the levels of ADA in mice treated with Maxatezo were significantly lower compared with animals treated with Atezolizumab. Most importantly, at the same dose (10 mg/kg), the tumor growth inhibition rate of Maxatezo was 98%, compared to 68% for Atezolizumab.

## Introduction

In recent years, with greater understanding of the mechanism of tumor immune escape and the tumor microenvironment, immune-checkpoint inhibitor-based therapies have shown satisfactory clinical efficacy in a variety of tumors. Immunotherapy plays an important role in the treatment of malignant tumors, and the use of anti-PD-1 and its ligand PD-Ll antibodies in the treatment of malignant tumors has become a focus of research. Several anti-PD-1 or PD-L1 antibodies have been approved by the FDA, such as Pembrolizumab (Merck), Nivolumab (BMS) and Atezolizumab (Roche). In 2019, while the sales of Pembrolizumab and Nivolumab reached $11.084 billion and $8.017 billion respectively, the sales of Atezolizumab were only $2.091 billion. One of reasons contributing to the under-performance of Atezolizumab may be its high ADA rates in cancer patients. Quite a few Phase III clinical tries of Atezolizumab did not reach the clinical end point (in 2017, IMvigor211 for advanced bladder cancer failed in clinical Phase III; in 2018, IMblaze370 for colorectal cancer failed in clinical Phase III; in 2019, IMspire170 for melanoma failed in clinical Phase III)^1^.

It is well-known in antibody manufacturing that incomplete glycosylation will lead to aggregations of antibodies^2^, which in turn may induce ADA^3^. Aglycosylation of antibody made the cases even worse. Atezolizumab is an aglycosylated human IgG1. In its pre-clinical study, 100% of Atezolizumab-treated monkeys developed strong ADA. In clinical studies, even though their immune systems had been severely damaged by chemo or radiotherapy, 41.5% of the cancer patients developed ADA^4^. However, it should be noted that ADA was observed to have no clinically significant effect on the incidence or severity of adverse reactions^4^.

Therapeutic antibodies have different mechanisms of actions, including, but not limited to (1) neutralizing antibodies that block the target/pathogen’s interaction with their cellular receptors, (2) clearing antibodies that mediate action by antibody-dependent cell phagocytosis (ADCP) to remove the target/pathogen from the body, or (3) targeting antibodies via antibody-dependent cytotoxicity (ADCC) to recruit natural killer (NK) cells and other effector T cells to kill the pathogens or tumor cells. Based on the intended mechanisms of action, researchers and drug developers may choose IgG1, IgG2 and IgG4 isotypes for their antibody drugs^5,6^, while IgG3 is avoided due to its instability. The sequence homology between the Fc of IgG1, IgG2 and IgG4 is more than 90%, with the primary differences resting within the hinge region and CH2 domain, which contain the binding sites for different FcγRs^7,8^.

FcγR engagement is essential for the Fc functions of IgGs^9–11^. Once the Fv binds to antigen, the antibody molecule will change the conformation of its Fc region to expose the binding sites for FcγRs, which in turn can activate ADCC and/or ADCP activity^12,13^. The human FcγR family consists of the activating receptors FcγRI, FcγRIIA, and FcγRIIIA, and the inhibitory receptor FcγRIIB^14^. FcγRI and FcγRIIA are expressed by macrophages, and involved in ADCP function^15,16^, while FcγRIIIA is expressed on NK cells and is important for ADCC function^17^.

For some antibodies targeting tumor-specific antigens on the surfaces of cancer cells, ADCC mediated effects allow NK cells to effectively kill tumor cells^18^. For development of these types of therapeutic antibodies, IgG1 isotypes with strong ADCC functions are preferred, and some of the antibodies are even modified to further enhance their ADCC effects^19^.

For blocking antibodies, such as antibodies targeting soluble cytokines (TNF alpha and IL17A) or some immune checkpoints (CTLA-4, PD-1, PD-L1) which are not tumor-specific, the cytotoxicity brought about by ADCC/ADCP should be avoided. Pembrolizumab and Nivolumab used IgG4, which are weak in ADCC. The FDA-approved anti-PD-L1 antibody drug Atezolizumab is a human IgG1 without glycosylation through a N297A mutation.

The binding affinity of IgG1 to FcγRs is highly dependent on the N-linked glycan at asparagine 297 (N297) in its CH2 domain^20,21^. Loss of binding to FcγRs was observed in (1) IgG1 with N297A point mutants^22,23^, (2) de-glycosylated IgG1 by enzymatic Fc deglycosylation^24^, (3) IgG1 expressed in the presence of the N-linked glycosylation-inhibitor tunicamycin^25^, or (4) expressed in bacteria^26,27^. In addition, the nature of the carbohydrate attached to N297 modulates the affinity of the FcγR as well^28,29^. Aglycosylation of IgG1 has been used to completely remove the un-wanted ADCC/CDC^23,30^.

Structures of the IgG1 Fc region show that the oligosaccharide attached to N297 is hidden in the cavity between the CH2 domains from the two heavy chains. When antibody binds to the antigen, there are conformation changes in the Fv regions, which result in a cascade of conformation changes, including the exposure of oligosaccharides attached to the N297 and the formation of the FcγR binding sites. The antigen-bound antibody is capable of binding the FcγR on the surfaces of effector cells, leading to ADCC and ADCP activation^21^. In theory, if one can block the conformation change, the signal transduction from the Fv to the Fc, this should also prevent the formation of the FcγR binding site, and thus prevent the ADCC/ADCP activation.

In this paper, we present a novel way to engineer antibody drugs without ADCC and other Fc functions by simply inserting a flexible amino acid sequence (GGGS) into the hinge region of the antibody’s Fc region. Our data shows that such an insertion completely abolishes the antibody’s ADCC. Effects on binding affinity (EC50), inhibitory activity (IC50), glycosylation profile, expression level, stability, immunogenicity and anti-tumor activity were also examined.

## Results

### Insertion of GGGS removed ADCC activity of anti-PD-L1 antibody with no negative impact on binding affinity and inhibitory activity of PD-L1

Based on the structural information acquired from different antibodies in the Protein Data Bank (e.g., PDB ID: 1IGT), we hypothesized that an insertion of a short but flexible amino acid sequence in the hinge region or somewhere upstream of the glycosylation site of N297 may mediate the stress transmission signal between the Fv and Fc domain. In this study, a short sequence of four amino acids, GGGS, was inserted between G237 and G238 of human IgG1 heavy chain. “GGGS” has been used in many approved biological drugs, such as scFv and Fc-fusion proteins, as flexible linkers without any known adverse effects or new immunogenicity in patients. For reverse engineering of Atezolizumab, a back mutation of A297N was also introduced into the original heavy chain to restore glycosylation (Fig. S1).

Genes encoding either the heavy chains of Atezolizumab or the modified version, Maxatezo, were chemically synthesized and ligated into expression vectors. Transient co-transfections of the light and heavy chains of IgG1 into CHO-K1 cells were carried out. The recombinant antibodies were purified from the culture supernatants and subjected to further characterizations.

**Figure 1:**
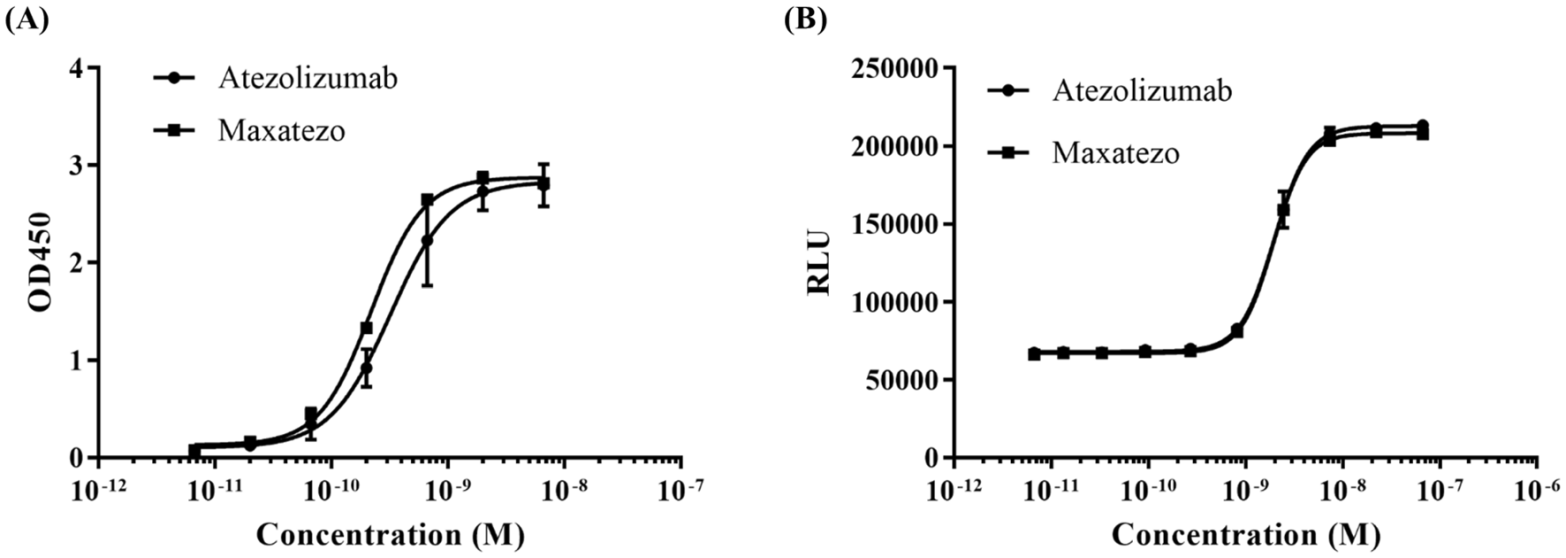
The Fc function removed the PD-L1 antibody, Maxatezo has the same binding affinity and inhibitory activity as Atezolizumab. (**A**) Binding affinities of Maxatezo and Atezolizumab, measured by Indirect ELISA against recombinant PD-L1. The square symbols represent Maxatezo, and the circular symbols represent Atezolizumab. The horizontal coordinate is antibody concentration and the vertical coordinate is absorbance value at 450 nm. (**B**). Inhibitory affinity evaluation of Maxatezo and Atezolizumab, measured by in vitro cell-based Bioassay using the Jurkat-PD-1-NFAT cell line that stably expresses PD-1 with the reporter luciferase gene. The square symbol represents Maxatezo, and the circular symbol represents Atezolizumab. The horizontal coordinate is antibody concentration and the vertical coordinate is mean value of the relative fluorescence units (RLU).

To examine whether there will be any negative impact on antibody affinity, we studied affinity to the recombinant PD-L1 using an indirect ELISA. As shown in Fig. 1A, both Atezolizumab and Maxatezo bound to PD-L1 in a dose-dependent manner, with almost identical EC50s, between 0.22 ~ 0.32 nM. Therefore, no negative impact on antibody affinity by such an insertion was observed.

Next, we examined the potential impact of the insertion of GGGS on inhibitory activities of anti-PD-L1 antibodies. The studies were carried out using an in vitro co-culture of a Jurkat-PD-1-NFAT cell line that stably expresses PD-1 with the reporter luciferase gene and CHO-PD-L1-CD3L cells that stably express PD-L1 and a membrane-anchored anti-CD3 single chain antibody fragment (scFv). The binding of PD-1 on the surface of Jurkat-PD-1-NFAT cells to the PD-L1 on the CHO-PD-L1-CD3L cells can block the downstream signal transduction of CD3 activated by the binding of anti-CD3 scFv, thus inhibiting the expression of luciferase in Jurkat-PD-1-NFAT cells. When PD-1 antibody or PD-L1 antibody is added, it will reverse the inhibition of luciferase expression. Addition of either Maxatezo or Atezolizumab reversed the inhibition of PD-1 on CD3-activated luciferase expressions in a dose-dependent manner, and both had an IC50 of 1.95 ~ 1.99 nM (Fig. 1B).

Clearly, the insertion of GGGS between G237 and G238 of human IgG1 heavy chain showed no significant negative impact on the antibody’s affinity and inhibitory activity.

We studied whether the insertion of GGGS removes the ADCC of human IgG1. Three anti-PD-L1 antibodies, IMM25 (ADCC-enhanced version produced by afucosylation of N297), Atezolizumab (de-ADCC version produced by N297A mutation) and Maxatezo (de-ADCC version produced by GGGS insertion) were tested in parallel for their ADCC activities using a co-culture cell killing assay in vitro. Briefly, 5(6)-Carboxyfluorescein diacetate N-succinimidyl ester (CFSE)-pre-dyed PD-L1-overexpressing Raji cells were mixed with FcR-overexpressing TANK cells in the presence or absence of PD-L1 antibodies. Once the PD-L1 antibody binds to the PD-L1 on the surface of the Raji cells, the FcR of the TANK cells will bind to the PD-L1-bound anti-PD-L1 antibody, which in turn will activate the releasing of cytotoxic cytokines from the TANK cells and eventually kill the Raji cells.

**Figure 2:**
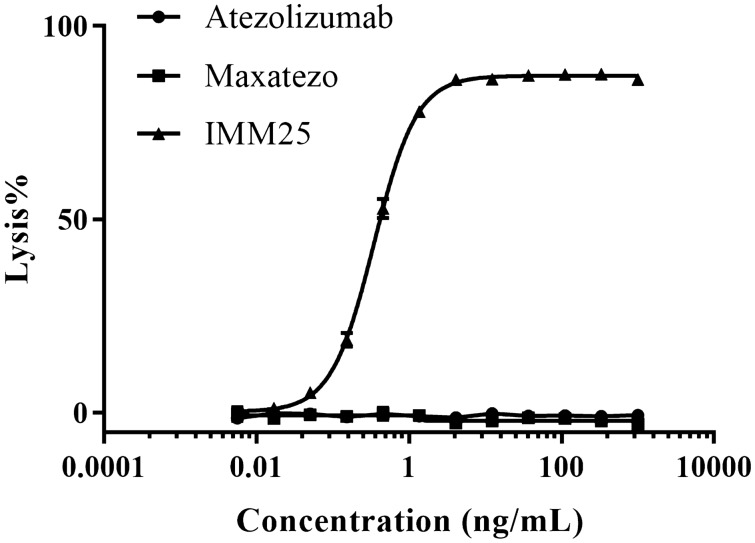
Insertion of GGGS removes ADCC activity completely. The ADCC activities of the different PD-L1 antibodies were measured by an in vitro cell killing assay using PD-L1-overexpressed Raji cells together with CD16a (158 V)-overexpressed NK92MI cells in the presence or absence of PD-L1 antibodies. The line with triangles represents the IMM25 (ADCC- enhanced anti-PD-L1 antibody), the line with squares represents the Maxatezo, (anti-PD-L1 antibody with GGGS insertion), the line with solid circles represents the Atezolizumab (anti-PD-L1 antibody with N297A). The horizontal coordinate is antibody concentration and the vertical coordinate is the percentage of ADCC induced cell lysis.

The positive control, an ADCC-enhanced anti-PD-L1 antibody (IMM25) had very strong ADCC activity in a dose-dependent manner, while both Maxatezo and Atezolizumab showed no ADCC activities at all (Fig. 2). This indicates that GGGS insertion in the Fc hinge region completely abolished the ADCC activity of human IgG1.

### Restoration of glycosylation improved the expression of anti-PD-L1 Maxatezo

**Figure 3:**
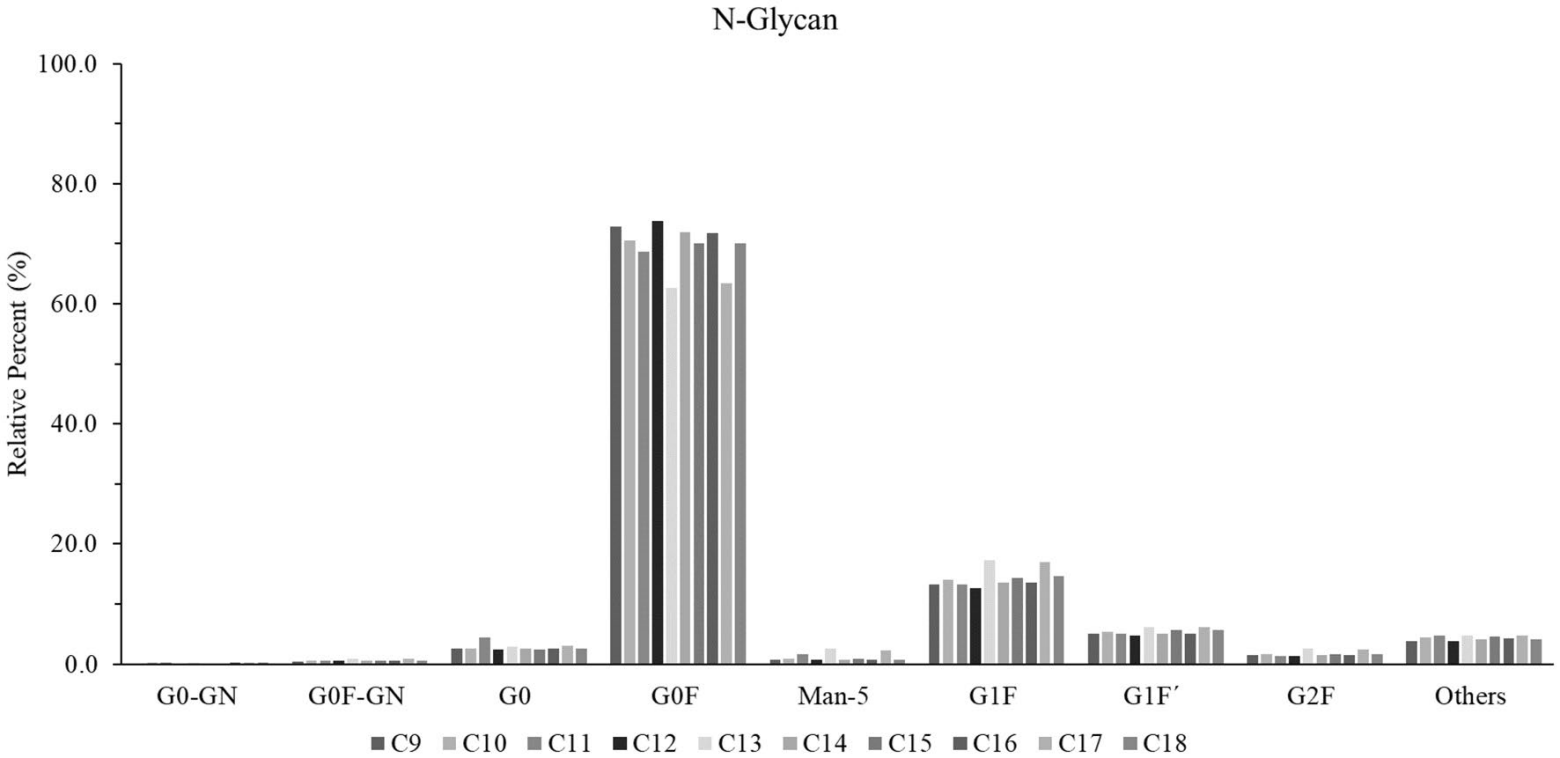
Insertion of GGGS does not affect the distribution of glycoform. The relative percentages of different glycans were examined. C9 to C18 represents different stable cell lines. *G* galactose, *F* fucose, *Man* mannose, *GN* N-acetylglucosamine. For example, G1F contains Fc oligosaccharides in which the core hepta-saccharide bears one terminal galactose moiety and also is fucosylated. The vertical coordinate represents relative percentages of different glycans.

Glycosylation is important for the correct conformation, secretion and stability of an antibody drug, and, in addition, its carbohydrate moiety also plays a great role in antibody affinity to FcγRs. Therefore, it is important to confirm that the insertion does not result in loss of glycosylation and/or significant change(s) to the glycan profile. Both Maxatezo and Atezolizumab were subjected to digestion with PNGase F to release the glycan from the antibodies. The enzymatic products were labeled with 2-Aminobenzamide (2-AB), then separated by Hydrophilic Interaction Chromatography (HILIC) with a fluorescence detector. While there was no glycan peak detected in the samples digested from Atezolizumab, two major peaks, (G0F and G1F), at retention times 19.888 and 22.561 were detected in the samples prepared from Maxatezo (Fig. S2). The percentages of different glycans of ten different clones of Maxatezo were examined. As shown in Table S1 and Fig. 3, G0F represented the majority (62–73%) of glycans, while G1F represented 12–17%. These data indicate that the insertion of GGGS does not change the glycan profile of the antibody drug.

For cost-effective therapeutic application, the expression level of recombinant an antibody drug in a mammalian cell system should be 2 g/L or higher. Firstly, we developed stable cell pools expressing the Atezolizumab or Maxatezo. Shown in Table S2, the expression levels of aglycosylated antibody were 1.3 g/L, while that of Maxatezo were up to 4 g/L. In another experiment, several production runs of using CHO cell lines which stably expressed Maxatezo were carried out. On day twelve, the culture supernatants were collected and the antibody concentrations in the culture supernatants were measured by HPLC. As shown in Table S3, the expression levels of Maxatezo in a stable CHO cell line were 4.73 ~ 5.45 g/L, meeting industry standards. After cloning, the expression level of Maxatezo by one of the CHO monoclonal cell lines reached 6.2 g/L. Clearly, insertion of GGGS does not have a negative impact on expression of the recombinant antibody.

### Glycosylated Maxatezo showed better thermostability and reduced immunogenicity

High molecular weight (HMW) aggregates in antibody drugs can induce anti-drug antibody (ADA)^3^, though this has not been directly observed for Atezolizumab. Aglycosylation of IgG1 can make the aggregation even worse. To examine the levels of aggregates in the final drugs, Maxatezo and Atezolizumab were separated by HPLC-SEC. The percentages of HMW aggregates in both antibody solutions were estimated to be less than 1.0%, based on the peak areas of monomer and HMW aggregates (Fig. S3, Table S4).

However, in data for the monomers, Atezolizumab had an absorbance of 21572737 units, 10% less than Maxatezo’s 23431856 units, The loading amounts were the same for both antibodies. We suspect that Atezolizumab may have significant amounts of invisible aggregates which were removed by the pre-filter of the HPLC columns.

**Figure 4:**
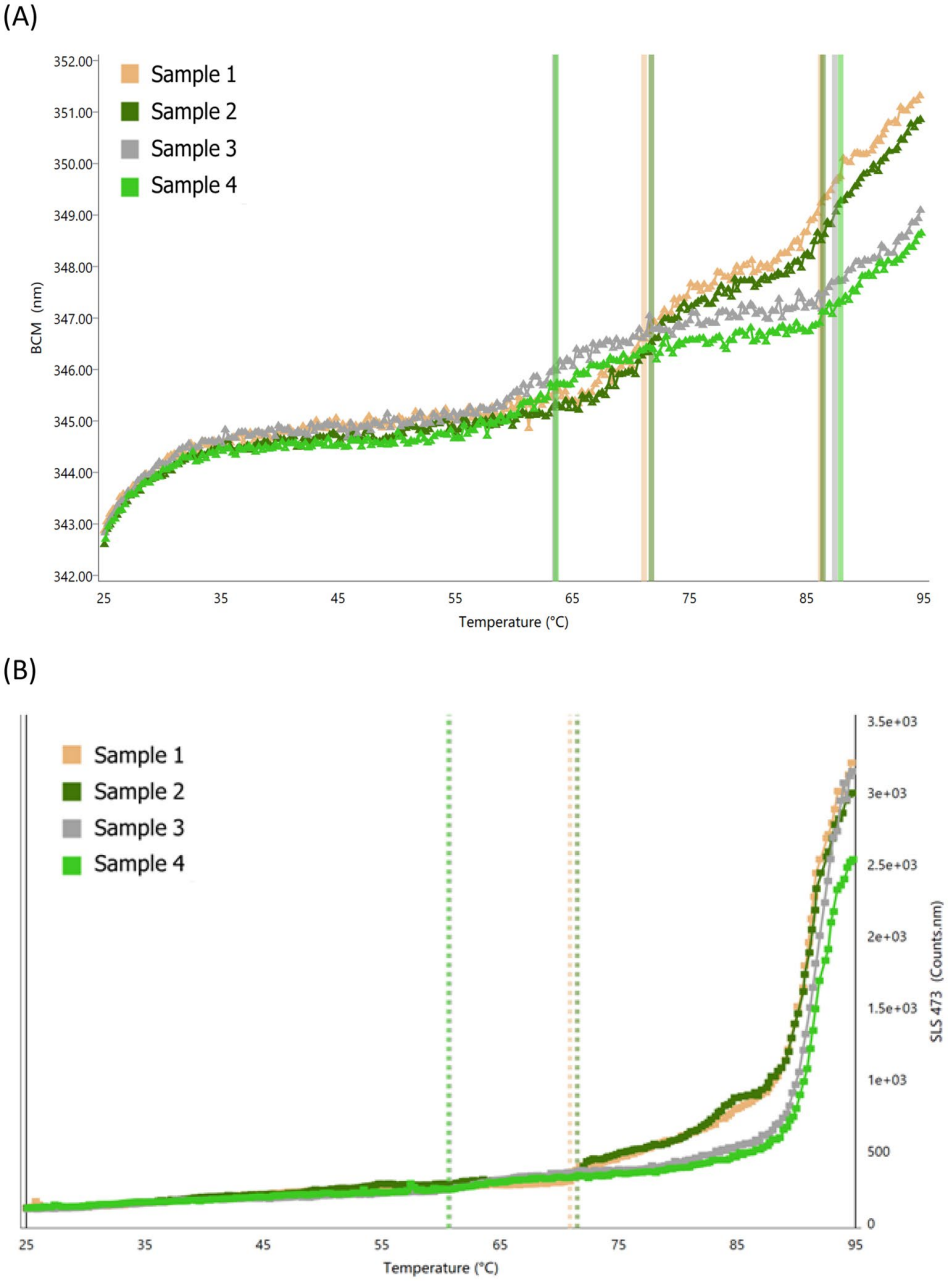
Maxatezo shows better thermostability than Atezolizumab. The stabilities of the antibodies were characterized by T_m_ and T_agg_. (**A**) T_m_ was obtained by intrinsic protein fluorescence (IPF) (266 nm excitation, 280–450 nm emission scan) and (**B**) T_agg_ w was monitored by SLS at 473 nm using the Uncle system (Unchained Labs). Duplicate samples of both Maxatezo and Atezolizumab were measured. The orange and dark green curves represent Maxatezo, and the gray and light green curves represent Atezolizumab.

To address this question, the thermal stabilities of Maxatezo and Atezolizumab were assessed by measuring Tm monitored by Intrinsic Protein Fluorescence (IPF) and the onset T_agg_ monitored by SLS (473 nm) using an Uncle system (Unchained Labs, Pleasanton, CA, see Methods). The label-free fluorescence from intrinsic aromatic amino acid residues excited by the inset excitation wavelength of 266 nm was determined for melting temperature (the midpoint of unfolding event, Tm) calculation. As shown in Table S5 and Fig. 4A, Maxatezo’s T_m1_ is 71.01 °C, which is 7.46 °C higher than that of Atezolizumab (63.55 °C), suggesting that the glycosylated Maxatezo is more stable than the aglycosylated Atezolizumab. Moreover, as shown in Table S4 and Fig. 4B, the T_agg_ of Maxatezo is 71.2 °C, which is 10.5 °C higher than that of Atezolizumab (60.7 °C), suggesting that Maxatezo will produce less aggregates than Atezolizumab.

In addition, the dynamic size distribution of the samples was determined by a Dynamic Light Scattering (DLS) module at 660 nm before the heating program (as shown in Table S5). The polydispersity index (PDI) of Atezolizumab was greater than 0.1, while that of Maxatezo was less than 0.1.

Based on above data, we concluded that the new De-ADCC anti-PD-L1 antibody Maxatezo was much more stable than Atezolizumab.

**Figure 5:**
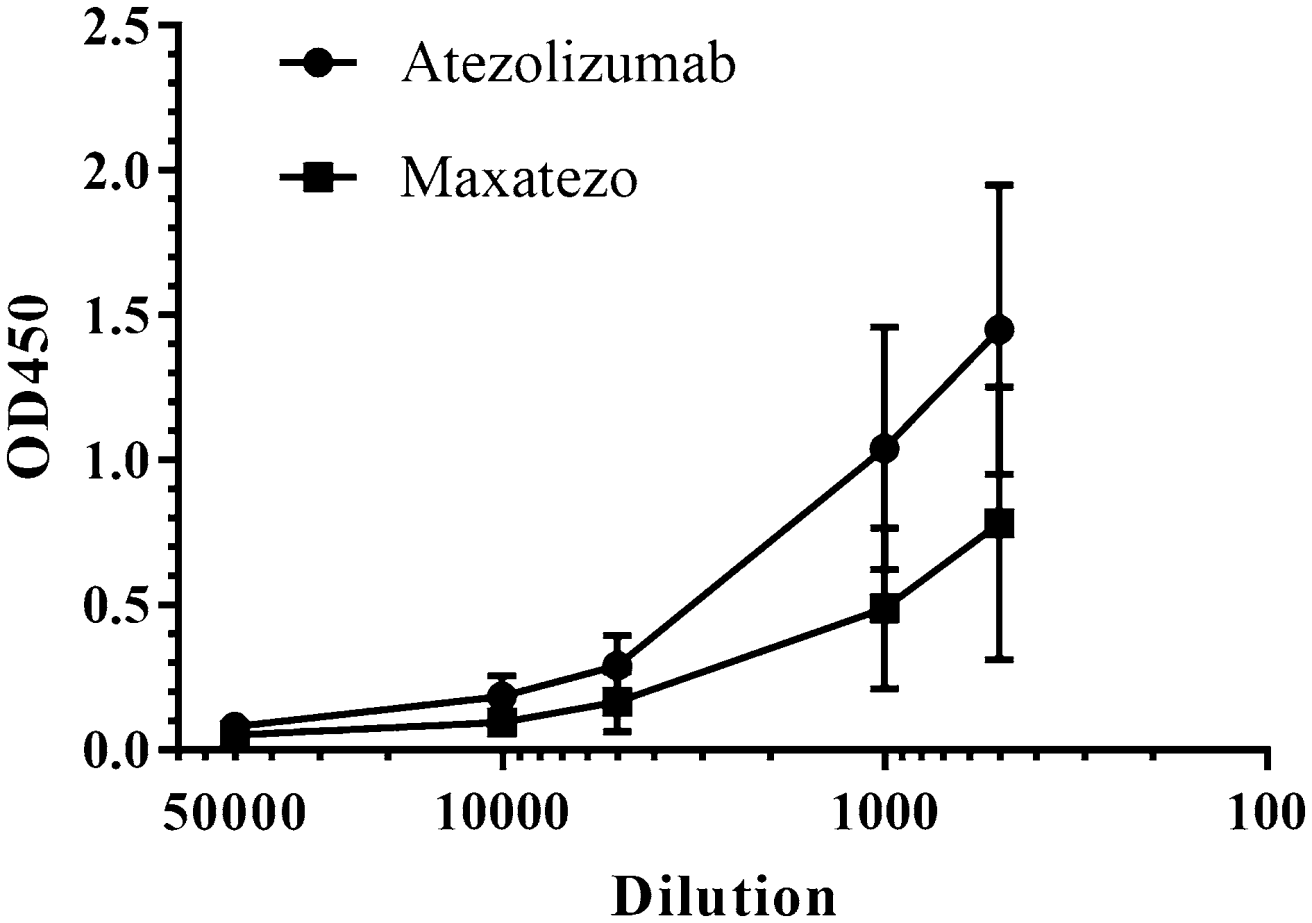
Maxatezo has lower immunogenicity than Atezolizumab in mice. Immunogenicity of antibodies was assessed using an in vivo mouse model (n = 5) and the ADA titers were measured using Indirect ELISA against antibody drugs. Data are presented as mean ± SD. The line with squares represents the Maxatezo, (anti-PD-L1 antibody with GGGS insertion), the line with circles represents the Atezolizumab (anti-PD-L1 antibody with N297A). The horizontal coordinate is the antibody dilution ratio and the vertical coordinate is the absorbance value at 450 nm.

In vivo immunogenicity assessment was carried out to compare the ADA titers in mice injected with either Atezolizumab or Maxatezo. As shown in Fig. 5, Atezolizumab induced high titers of ADA in mice, in good agreement with what was observed in monkeys (100%) and cancer patients (41.5%). The ADA titers of Maxatezo in mice was significantly less than that of the aglycosylated Atezolizumab. Since both Atezolizumab and Maxatezo have almost identical humanized mouse antibody sequences except the insertion of GGGS and mutation N297A, we think that the differences of immunogenicity we observed in mice is due to the difference of aggregation levels in the antibody drugs.

### Maxatezo demonstrated better tumor growth inhibition than Aezolizumab in the MC38 mouse tumor model

**Figure 6:**
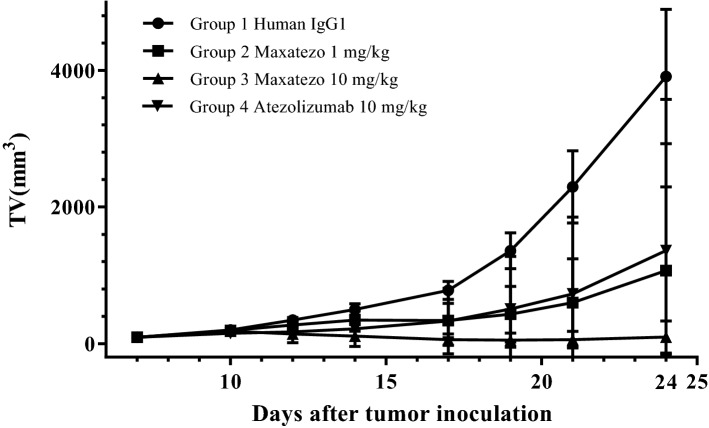
Maxatezo has stronger anti- tumor activity in vivo*.* MC38 cells were injected subcutaneously into C57BL/6 mice (n = 8). On day 7, the animals were randomly grouped and treated with Maxatezo (1 mg/kg, 10 mg/kg), Atezolizumab (10 mg/kg) and human IgG1 a control every 3 days. The plots show changes in tumor volume over time in Maxatezo-treated versus Atezolizumab-treated mice. The data are presented as mean ± SD. The horizontal coordinate represents the days after tumor inoculation and the vertical coordinate represents the tumor volume (mm^3^).

To examine the tumor growth inhibitory activity of the Anti-PD-L1 antibody drug, mice were first injected subcutaneously with mouse colorectal tumor MC38 cells. On day 7, the animals were randomly grouped into four groups with 8 mice per group. Placebo or antibody drugs were administered to the mice according to the predetermined regimen shown in Table S6. The tumor volumes were measured twice weekly. As shown in Fig. 6 and Table S7. in this experiment with a syngeneic mouse colon cancer model, Maxatezo and Atezolizumab significantly inhibited the growth of subcutaneously grafted MC38 tumors in C57BL/6 mice, compared with the mice treated with isotype control antibody human IgG1. A dose-dependent inhibition of tumor volumes was observed with Maxatezo between 1 mg/kg and 10 mg/kg. At a dose of 10 mg/kg, Maxatezo demonstrated better tumor growth inhibition than Atezolizumab.

At end of the study on day 24, animals were terminated and tumors were collected, photographed and weighed. As shown in Fig. S4, tumors in anti-PD-L1 antibody-treated groups were all significantly smaller than those in the human IgG1 control group. Maxatezo treatment at 10 mg/kg resulted in significantly smaller tumors compared with Maxatezo at 1 mg/kg and Atezolizumab at 10 mg/kg. At 10 mg/kg, complete tumor regression was observed in 5 out of 8 mice treated with Maxatezo, while complete tumor regression was seen in 4 mice in the Atezolizumab group. Most significantly, the tumor sizes were much smaller in the Maxatezo-treated group.

Tumor weight inhibition (TWI %) was calculated as (1-average tumor weight of each drug-treated group/average TW of the control group) × 100%. As summarized in Table S8, Maxatezo (1 mg/kg), Maxatezo (10 mg/kg), and Atezolizumab (10 mg/kg) treatments produced TWI values of 74%, 98% and 65%, respectively. Tumor-bearing mice showed good tolerance to continuous administration of Maxatezo and Atezolizumab in this experiment.

Our data demonstrates that the insertion of GGGS in the hinge regions of human IgG1 could abolish ADCC activities completely without negative impact on antibody affinities and inhibitory activities. Restoration of glycosylation actually improved the expression level and stability of anti-PD-L1 antibody, and reduced its immunogenicity. Most importantly, Maxatezo showed much stronger anti-tumor activity than its aglycosylated analogue.

## Discussion

Immunotherapy using monoclonal antibodies against PD1, PD-L1 or CTLA-4, has been demonstrated to be effective to treat various cancers. Therapeutic fields for antibody drugs also expand beyond autoimmune disease, cancers and infectious diseases into chronic diseases such as pain, neurodegenerations, diabetes, and osteoporosis. To reduce the immune-related adverse effects (irAE), switching IgG1 to IgG2/IgG4 or use of aglycosylation of IgG1 has been widely employed to remove the Fc function(s) of antibody drugs. However, IgG2 has an extra cystine residue in the upstream of the hinge region and will form homo- or hetero- dimers via inter-IgG2 disulfide bonds, which will in turn affect antibody expression level and stability^31^. IgG4 has reduced ADCC but retains ADCP activity. It is believed that ADCP activity of Pembrolizumab may reduce its tumor killing potency by phagocytosis of anti-PD-1 antibody-activated NK cells^32^.

A new Fc function removal technology based on structural biology is reported in this paper. A flexible sequence, (in this case, GGGS), was inserted into the hinge region of IgG1 isotype antibody to interrupt the stress signal transfer between the Fv and Fc region. Because of this, the FcγR binding domain will not be exposed when the antibody binds to the target, leading to the loss of ADCC activity.

The rational design of human IgG1 antibody lacking ADCC or other Fc functions is a very promising approach to develop therapeutic antibodies without an antibody’s unwanted Fc functions. For those well-known antibody drugs, such as Genentech’s aglycosylated anti-PD-L1 Atezolizumab, we demonstrated that inserting GGGS in the hinge regions of human IgG1 Fc could remove the ADCC activities completely. Since this approach does not alter the Fv domain of the antibody, we did not observe a negative impact on either the affinity or inhibitory activity.

The study strategized a physics-based approach to solve a problem of biology. Inspired by previous studies demonstrating the structural change of the Fc hinge region after the binding of antigens, we simply inserted a flexible linker to stop the stress transmission of the antibody from Fv to Fc, preventing the exposure of binding sites for various FcγRs. Leaving glycosylation intact and making no additional changes in the remaining parts of the Fc region not only resulted in much higher expression levels than the aglycosylated Atzeolizumab, but stability was also improved. We feel that Maxatezo, the re-engineered anti-PD-L1, is ready to proceed into pre-clinical studies.

As of June 2020, there are 10 antibody drugs on the market and more than 50 in clinical trials with purposely reduced ADCC and/or ADCP activities. In a separate study, using the same De-Fc technology, we have successfully re-engineered anti-PD-1 antibody (Pembrolizumab), anti-CTLA-4 antibody (Ipilimumab) and anti-CD47 antibody (Magrolimab) without ADCC and/or ADCP (data not shown). The De-Fc function method could be universal. It may offer a better way to develop safer antibody drugs with less irAE or improve the efficacy of antibody drugs. Thus, the prospects of this Fc function removal technology is promising.

Furthermore, in SARS-CoV infection patients, the anti-spike IgG causes severe acute lung injury through the FcγRs, which could skew alveolar macrophages from wound-healing to proinflammatory^33^ responses. We expect that our technology can also help develop COVID-19 therapeutic antibodies with much less proinflammatory activities.

## Materials and methods

### Cell lines and reagents

AxyPrep DNA Gel Extraction Kit (Cat: AP-GX-250) and AxyPrep Plasmid Miniprep Kit (Cat: AP-MN-P-250) were purchased from Axygen (NY, USA). T4 DNA ligase (Cat: M0202) was from NEB (MA, USA), and yeast extract (Cat: LP0021) and tryptone (Cat: LP0042) were from Oxoid (MA, USA). CHO-K1 cells and serum-free medium for transient transfection (KD-CHO, Cat: K03201) were obtained from Zhuhai Kairui Biotech, Ltd (Zhuhai, China). The stable cell pool was constructed by Zhenge Biotechnology Co. (Shanghai, China). For stable cell pool construction, the adherent CHO K1 parental cells were obtained from ATCC (VA, USA). After adapted into suspended and serum-free culture, the CHO K1 cells were cultured in CD CHO medium (Cat: 10743002) from Gibco (CA, USA). The viable and total cell counts were determined by a Vi-CELL XR Cell Counter from Beckman (FL, USA). Atezolizumab and Maxatezo were produced from stable cells by Zhenge Biotechnology Co. (Shanghai, China). Complete Freund's adjuvant (CFA, Cat: F5881), incomplete Freund's adjuvant (IFA, Cat: F5506), PI fluorochrome (Cat: P4170), CFSE (5(6)-Carboxyfluorescein diacetate N-succinimidyl ester, Cat: 21888) and TMB substrates (Cat: T8665) were purchased from SIGMA (MO, USA). Recombinant PD-L1 (Cat: 10084-H08H) was purchased from Sino Biological (Beijing, China). Peroxidase AffiniPure Goat Anti-Mouse IgG (subclasses 1 + 2a + 2b + 3), Fcγ Fragment Specific (Cat: 115-035-164) and Peroxidase AffiniPure Goat Anti-Human IgG(H + L) (Cat: 109–035-003) were from Jackson Immune Lab (PA, USA). The CHO-PD-L1-CD3L and Jurkat-PD-1-NFAT cell lines were provided by the National Institutes for Food and Drug Control (NIFDC) (Beijing, China). The CD16a (158 V)-overexpressed NK92MI cells (FcR-TANK cells), and PD-L1-overexpressing Raji cells were provided by ImmuneOnco Biopharma Co., Ltd (Shanghai, China). The melting temperature (T_m_) and aggregation temperature (T_agg_) of the antibodies were measured using an Uncle system (Unchained Labs, Pleasanton, CA). Female BALB/c mice were obtained from Vital River Co. (Beijing, China). The C57BL/6J mice were obtained from GemPharmatech Co., Ltd (Changzhou, China). The MC38 cell line was purchased from Biovector NTCC Inc. (Beijing, China).

### Antibody production

The antibodies were expressed either by transient transfection of CHO-K1 cells or using a stable CHO-K1 cell pool and purified with Protein A Sepharose.

The heavy chain of PD-L1 antibody was constructed into the pEE12.4 plasmid, while the light chain of antibody was constructed into the pEE6.4 plasmid. For PD-L1 antibody transient expression, CHO-K1 cells were co-transfected with the plasmids containing either heavy chain or light chain at a mass ratio of 1:1. The transfected cells were cultured at 37 °C, 5% CO_2_ in shaking flasks at 120 rpm. The culture supernatants were collected on day 6 and the cell debris were removed by centrifugation at 3000 rpm.

To establish a stable cell pool expressing anti-PD-L1 antibody, after the transfection CHO-K1 cells were subjected to selection pressure with MTX at various concentrations, and the antibody expression levels by those mini-stable cell pools were evaluated. To obtain the stable monostable cell line, sub-cloning by limiting dilution was carried out from the selected stable mini-pools. The 3 most promising clones were selected for further development. The antibodies were purified from the culture supernatants using a Protein A Sepharose column.

### Indirect ELISA

The binding affinity of anti-PD-L1 antibody was determined by Indirect ELISA against the recombinant PD-L1. Each well of 96-well high binding EIA plates was coated with 1 µg/mL of PD-L1 at 4 °C overnight in PBS. After two washes with PBS and blocking with 5% skim milk in PBS for 1 h at room temperature, wells were incubated with purified PD-L1 antibodies in 5% skim milk in PBS for another hour at room temperature. After two washes with PBS, wells were then incubated with HRP-conjugated goat anti-human IgG Fc-specific secondary antibodies (Jackson Lab) in 5% skim milk in PBS for 1 h at room temperature. After five washes with PBS plus 0.1% Tween20 (PBST), the HRP substrate 3, 3´, 5, 5´-tetramethylbenzidine (TMB) solution was added. The reaction was stopped with 0.1 M H_2_SO_4_ after 30 min of incubation and absorbance was measured at 450 nm with a microplate reader.

### Bioassay of anti-PD-L1 antibodies

As reported previously^34^, two cell lines were used for this assay: (1) the CHO-PD-L1-CD3L cell line that stably over-expresses both PD-L1 and a membrane-anchored anti-CD3 single chain antibody fragment (scFv), and (2) the Jurkat-PD-1-NFAT cell line that stably over-expresses both PD-1 and the reporter luciferase gene under the control of the NFAT response elements from the IL-2 promoter.

Briefly, Jurkat-PD-1-NFAT cells were added to wells cultured with CHO-PD-L1-CD3L cells. The binding of PD-1 on the surface of Jurkat-PD-1-NFAT cells to the PD-L1 on the CHO-PD-L1-CD3L cells can block the downstream signal transduction of CD3 activated by the binding of anti-CD3 scFv, thus inhibiting the expression of luciferase in Jurkat-PD-1-NFAT cells. When PD-1 antibody or PD-L1 antibody is added, a reverse the inhibition of luciferase expression is seen.

CHO-PD-L1-CD3L cells were seeded at 50,000 cells per well in 96 well plates, incubated at 37 °C, with 5% CO_2_ for 12–14 h. 100,000 of Jurkat-PD-1-NFAT cells were added to each well in the presence or absence of PD-L1 antibody and incubated at 37 °C with 5% CO2 for another 6 h. Cells were lysed and 100 μL of luciferase substrate (Promega Bio-Glo Luciferase Assay) was added to each well, and the plate was measured using SpectraMax M5 to calculate the relative luciferase unit.

### In vitro ADCC detection assay

The ADCC activity of the antibody was evaluated by lysis of the PD-L1-overexpressing Raji cells (PD-L1-Raji) by the CD16a (158 V)-overexpressing NK92MI cells (FcR-TANK) in the presence of the anti-PD-L1 antibodies.

Briefly, PD-L1-Raji cells were first dyed with CFSE, then the concentration of the PD-L1-Raji cells adjusted to 6 × 10^5^/mL. The same concentration of FcR-TANK cells was also prepared. Serial dilutions of anti-PD-L1 antibodies, Maxatezo, Atezolizumab and IMM25 (the ADCC-enhanced PD-L1 antibody from ImmuneOnco Biopharma) were made with culture medium.

To each well of the 96-well culture plate, 50 μL of antibody solution, 50 μL of PD-L1-Raji cells and 100 μL of FcR-TANK cells were added. As a blank control, no antibody was added to the well. The plate was incubated at 37 °C with 5% CO_2_ for 4 h. At the end of the incubation, 5 μg/mL PI was added to stain the dying Raji cells. The populations of PI/CFSE-positive cells were analyzed using a flow-cytometer. For calculation of ADCC activity: Lysis% = [(Sample % PI Positive cell – Control % PI Positive cell) / (100 – Control % PI Positive cell)] × 100.

### Thermal stability assessment

The melting temperature (T_m_) and aggregation temperature (T_agg_) of the antibodies were measured using an Uncle system (Unchained Labs, Pleasanton, CA). T_m_ was determined by monitoring Intrinsic protein fluorescence (IPF) (266 nm excitation, 280–450 nm emission scan) and the T_agg_ was monitored by static light scattering (SLS) at 473 nm. Antibody solutions at 60 mg/mL were heated from 25 to 95 °C using 1 °C increments, with an equilibration time of 1 min before each measurement. Measurements were done in duplicate, and standard errors were calculated using GraphPad Prism 7.

### HPLC

For HPLC-SEC analysis of antibodies, the sample was diluted to 2.0 mg/mL with the mobile phase (50 mM NaH_2_PO4, 300 mM NaCl, pH 7.0), then 50 μL loaded to the TSK gel G3000SWXL (5 μm, 7.8 × 300 mm) column which previously had been equilibrated with the same mobile phase. The procedure was then run with a 0.5 ml/min flow rate for 90 min. The absorbance value at 280 nm was monitored.

### Glycan assay

Antibodies were first digested with PNGase F from NNB according to the manufacturer’s instructions, and the free glycan(s) were separated using a Ludger Clean EB10 kit according to the manufacturer’s instructions. The glycan samples were labeled with 2-AB (2-aminobenzamide) using a Ludger Tag 2-AB (2-aminobenzamide) Glycan Labeling Kit and separated with Ludger Clean S cartridges. The 2-AB labeled glycan samples were analyzed by HPLC with fluorescence detection.

### In vivo immunogenicity assay

Balb/c mice were used to evaluate the ADA titers of the different antibodies. Five mice per group were first immunized with 70 μg antibodies in Complete Freund’s Adjuvant and boosted with 70 μg antibodies in Incomplete Freund’s Adjuvant four times in two weeks. Four weeks after the first immunization, tail bleeds from the antibody-immunized mice were tested for ADA titers by indirect Enzyme-linked Immunoassay (ELISA). Briefly, each well of the 96-well high binding EIA plates was coated with 1 µg/mL of Atezolizumab or Maxatezo at 4 °C overnight in PBS. The serum samples were first incubated with 1% human sera for one hour at room temperature to block nonspecific mouse anti-human IgG antibodies. After two washes with PBS and blocking with 5% skim milk in PBS for 1 h at room temperature, wells were incubated with pretreated serum in 5% skim milk in PBS for another hour at room temperature. After two washes with PBS, wells were then incubated with HRP-conjugated goat anti-mouse IgG Fc-specific secondary antibodies (Jackson Lab) in 5% skim milk in PBS for 1 h at room temperature. After five washes with PBS containing 0.1% Tween 20 (PBST), HRP substrate 3, 3´, 5, 5´-tetramethylbenzidine (TMB) solution was added. The reaction was stopped with 0.1 M H_2_SO_4_ after 30 min and absorbance was measured at 450 nm with a microplate reader.

### In vivo efficacy study of Maxatezo and Atezolizumab in MC38 mouse colorectal cancer model

The MC38 cell line was purchased from Biovector NTCC Inc. The tumor cell was maintained in vitro in DMEM medium supplemented with 10% heat-inactivated fetal calf serum, 100U/ml penicillin and 100 μg/ml streptomycin at 37 °C in 5% CO_2_. The cells growing in exponential phase were harvested and suspended in PBS at 1 × 10^7^/ml. 100 µl of the MC38 cells were subcutaneously inoculated in the right flank of C57BL/6J mice. When the average tumor volume reached about 93 mm^3^, the mice were randomly grouped and antibodies were administered to the mice according to the regimen shown in Table S6.

After inoculation, the animals were checked daily for morbidity and mortality. At the time of routine monitoring, the animals were checked for any effects of tumor growth and treatment on normal behavior such as mobility, food and water consumption, body weight gain/loss, eye/hair matting and any other abnormal effect. Animal death and observable clinical signs were recorded.

The tumor volume was calculated according to the formula: tumor volume = 0.5 × long diameter × short diameter^2^, and the tumors were measured twice weekly. The T/C values were calculated from the tumor volume, where T is the average relative tumor volume (RTV) of each test subject treated group, and C is the average RTV of the control group. RTV is the ratio of tumor volume after administration pre-dose. Tumor growth inhibition (TGI%) was calculated as (1-T/C) × 100%. The treatments with tumor growth inhibition ≥ 60% and statistically significant difference in tumor volume were considered effective.

At the end of study, the animals were terminated and the tumors were collected, photographed, weighed and tumor weight inhibition (%) was calculated as (1-average TW of each test subject treated group/average TW of the control group) × 100%.

### Ethical statement

All studies were carried out in accordance with the ARRIVE guidelines, and all experimental procedures involving mice in this study were in accordance with requirements and guidelines for treatment of experimental animals that were set forth and approved by the National Institutes for Food and Drug Control of China. The Ethics Committee on animal experiments of AbMax Biotechnology has approved all animal experiments conducted in this manuscript.

## Supplementary Information

Supplementary Information
