# Author Correction: Next generation of anti-PD-L1 Atezolizumab with enhanced anti-tumor efficacy in vivo

**DOI:** 10.1038/s41598-022-09081-4

**Published:** 2022-03-28

**Authors:** Maohua Li, Rongqing Zhao, Jianxin Chen, Wenzhi Tian, Chenxi Xia, Xudong Liu, Yingzi Li, Song Li, Hunter Sun, Tong Shen, Wenlin Ren, Le Sun

**Affiliations:** 1 AbMax BioPharmaceuticals Co., LTD, 99 Kechuang 14th Street, BDA, Beijing, 101111 China; 2 AnyGo Technology Co., LTD, Beijing, China; 3 ZhenGe Biotechnology Co., LTD, Shanghai, China; 4 ImmuneOnco Biopharma (Shanghai) Co., LTD, Shanghai, China

Correction to: *Scientific Reports* 10.1038/s41598-021-85329-9, published online 11 March 2021

The original version of this Article contained errors in the discussion of Atezolizumab aggregation, and anti-drug antibody development.

In the Abstract section,

"However, aglycosylated Atezolizumab is very unstable and easy to form aggregation, which causes quick development of anti-drug antibody (ADA) in 41% of Atezolizumab-treated cancer patients, eventually leading to loss of efficacy"

now reads:

"However, aglycosylated Atezolizumab is unstable and easy to form aggregates."

In the Introduction section,

“One of reasons contributing to the under-performance of Atezolizumab may be its high ADA rates in cancer patients. Quite a few Phase III clinical tries of Atezolizumab did not reach the clinical end point (in 2017, IMvigor211 for advanced bladder cancer failed in clinical Phase III; in 2018, IMblaze370 for colorectal cancer failed in clinical Phase III; in 2019, IMspire170 for melanoma failed in clinical Phase III)^1^.”

now reads:

“Quite a few Phase III clinical tries of Atezolizumab did not reach the clinical end point (in 2017, IMvigor211 for advanced bladder cancer failed in clinical Phase III; in 2018, IMblaze370 for colorectal cancer failed in clinical Phase III; in 2019, IMspire170 for melanoma failed in clinical Phase III)^1^.”

In addition, the text,

“It is well-known in antibody manufacturing that incomplete glycosylation will lead to aggregations of antibodies^2^, which in turn will induce strong ADA in treated patients^3^.”

now reads:

“It is well-known in antibody manufacturing that incomplete glycosylation will lead to aggregations of antibodies^2^, which in turn may induce ADA^3^.”

And the text,

“In its pre-clinical study, 100% of Atezolizumab-treated monkeys developed strong ADA. In clinical studies, even though their immune systems had been severely damaged by chemo or radiotherapy, 41.5% of the cancer patients developed ADA^4^. The fast development of neutralizing ADA forced the dosage of Atezolizumab to be escalated to a record high of 1200 mg per injection,^4^ and still failed to reach end-points in several Phase III clinic trials^1^.”

now reads:

“In its pre-clinical study, 100% of Atezolizumab-treated monkeys developed strong ADA. In clinical studies, even though their immune systems had been severely damaged by chemo or radiotherapy, 41.5% of the cancer patients developed ADA^4^. However, it should be noted that ADA was observed to have no clinically significant effect on the incidence or severity of adverse reactions^4^.”

In the Results section:

"High molecular weight (HMW) aggregates in antibody drugs are the major causes of anti-drug antibody (ADA)^3^"

now reads:

"High molecular weight (HMW) aggregates in antibody drugs can induce anti-drug antibody (ADA)^3^, though this has not been directly observed for Atezolizumab."

In addition, the text,

“Moreover, as shown in Table S4 and Fig. 4B, the T_agg_ of Maxatezo is 71.2 °C, which is 10.5 °C higher than that of Atezolizumab (60.7 °C), indicating that Maxatezo produced much less aggregates than Atezolizumab.”

now reads:

“Moreover, as shown in Table S4 and Fig. 4B, the T_agg_ of Maxatezo is 71.2 °C, which is 10.5 °C higher than that of Atezolizumab (60.7 °C), suggesting that Maxatezo will produce less aggregates than Atezolizumab.”

And the text,

“As shown in Fig. 5, Atezolizumab induced extremely high titers of ADA in mice, in good agreement with what was observed in monkeys (100%) and cancer patients (41.5%). The ADA titers of Maxatezo in mice was significantly less than that of the aglycosylated Atezolizumab. Since both Atezolizumab and Maxatezo have almost identical humanized mouse antibody sequences except the insertion of GGGS and mutation N297A, we believe that the great differences of immunogenicity we observed in mice is due to the difference of aggregation levels in the antibody drugs, and we expect to see a significant reduction of ADA in Maxatezo-treated human patients.”

now reads:

“As shown in Fig. 5, Atezolizumab induced high titers of ADA in mice, in good agreement with what was observed in monkeys (100%) and cancer patients (41.5%). The ADA titers of Maxatezo in mice was significantly less than that of the aglycosylated Atezolizumab. Since both Atezolizumab and Maxatezo have almost identical humanized mouse antibody sequences except the insertion of GGGS and mutation N297A, we think that the differences of immunogenicity we observed in mice is due to the difference of aggregation levels in the antibody drugs.”

The original Article has been corrected.

