# Supplementary material for: Next generation of anti-PD-L1 Atezolizumab with enhanced anti-tumor efficacy in vivo
Source: Sci Rep. 2021 Mar 11;11:5774. doi: 10.1038/s41598-021-85329-9 (PMC7952408; doi:10.1038/s41598-021-85329-9)
Supplement: Supplementary file 1 — Supplementary Information [file 41598_2021_85329_MOESM1_ESM.docx]

**Title：Next generation of anti-PD-L1 Atezolizumab with enhanced anti-tumor efficacy *in vivo***

Maohua Li^1^, Rongqing Zhao^2^, Jianxin Chen^3^, Wenzhi Tian^4^, Chenxi Xia^1^, Xudong Liu^1^, Yingzi Li^1^, Song Li^4^, Hunter Sun^2^, Tong Shen^1^, Wenlin Ren^1 *^, Le Sun^1 *^

1. AbMax BioPharmaceuticals Co., LTD, Beijing, China
2. AnyGo Technology Co., LTD, Beijing, China
3. ZhenGe Biotechnology Co., LTD, Shanghai, China
4. ImmuneOnco Biopharma (Shanghai) Co., LTD, Shanghai, China

^*^Corresponding Authors:

Le Sun

AbMax BioPharmaceuticals Co., LTD, 99 Kechuang 14^th^ Street, BDA, Beijing, China 101111.

Phone: 86-10-59755729

Fax: 86-10-59755718

Email: sunl@antibodychina.com

Wenlin Ren

AbMax BioPharmaceuticals Co., LTD, 99 Kechuang 14^th^ Street, BDA, Beijing, China 101111.

Phone: 86-10-59755729

Fax: 86-10-59755718

Email: renwl@antibodychina.com

**
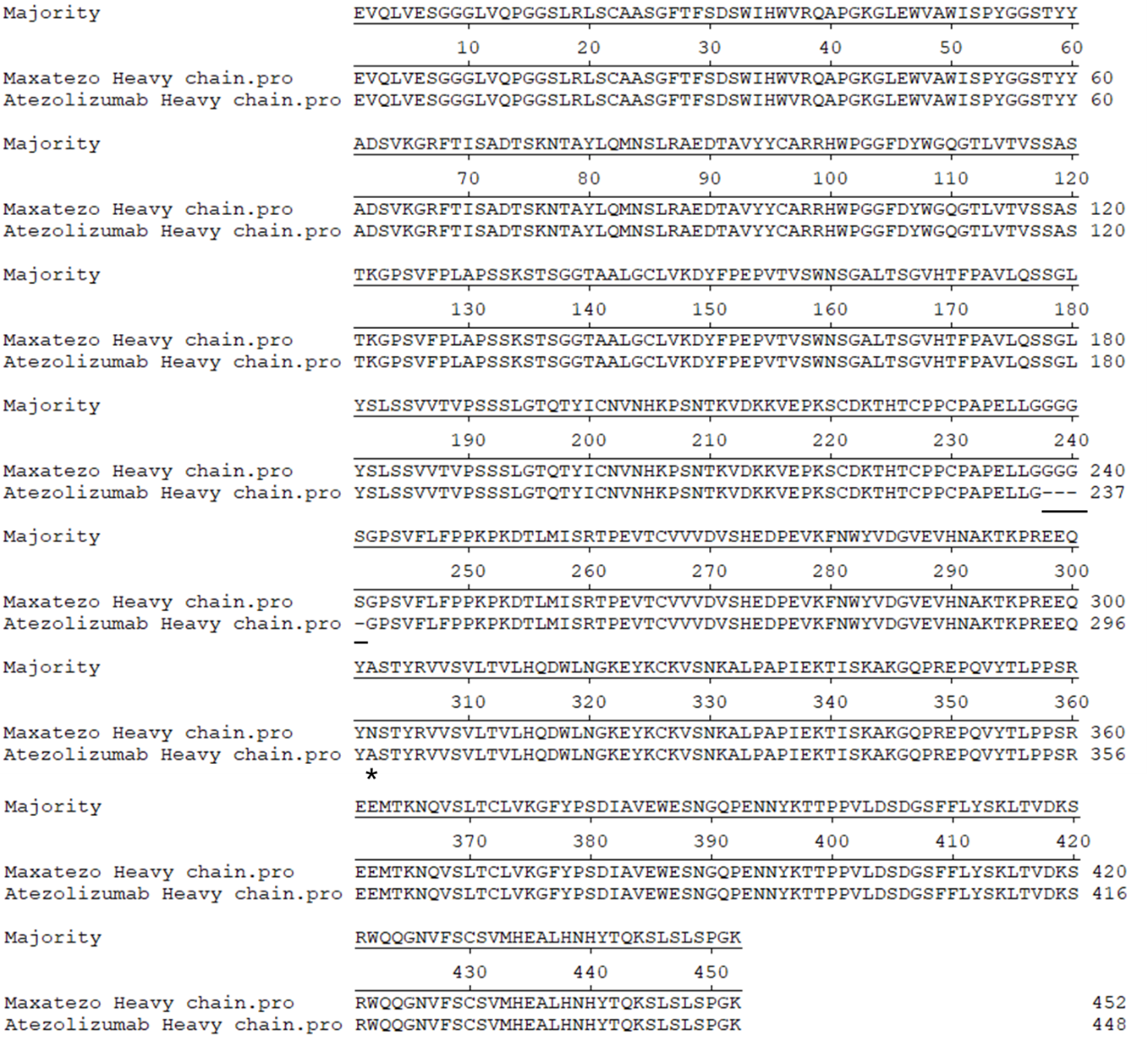
**

**Figure S1. Sequence alignment of Atezolizumab and Maxatezo**

(a). Sequence alignment of Atezolizumab and Maxatezo. The protein sequences of Atezolizumab and Maxatezo were aligned by the clustal W method. The insertion sequence and site are marked by an underline. The back mutation of A297N is indicated by *.

**Figure S2. Glycan analysis of anti-PD-L1 mAbs.**

. (A) The HILIC of Atezolizumab; (B) The HILIC of Maxatezo. The horizontal coordinate is retention time and the vertical coordinate is absorbance value at 280nm.

**
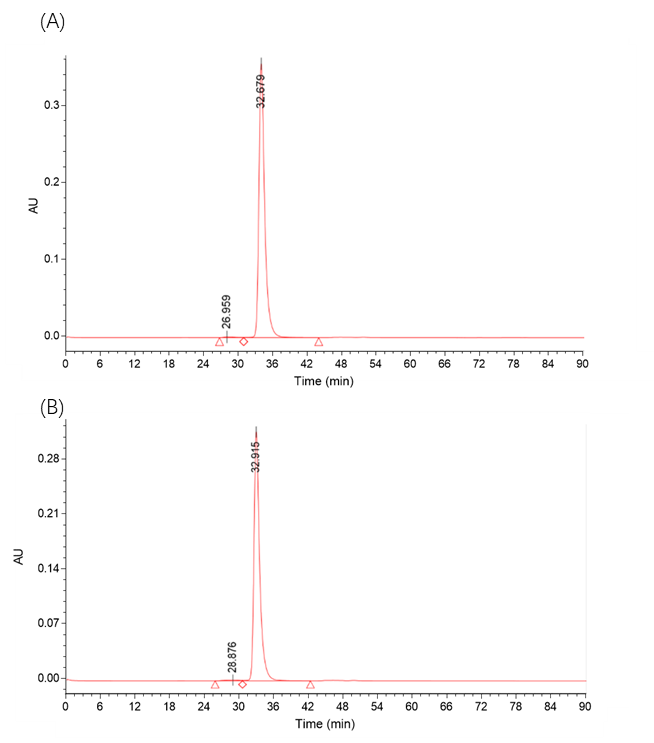
**

**Figure S3 Purity analysis of Maxatezo & Atezolizumab by HPLC-SEC.**

(A) Gel filtration chromatography of Maxatezo; (B) Gel filtration chromatography of Atezolizumab. The horizontal coordinate is retention time and the vertical coordinate is absorbance value at 280nm.


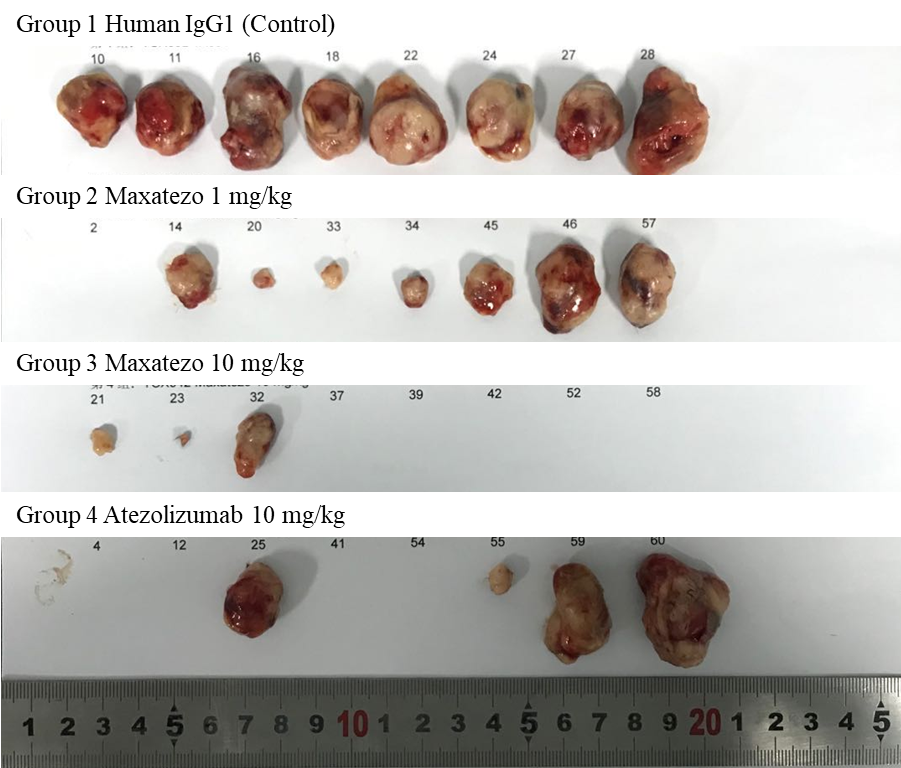


**Figure S4 Photographs of tumors**

The *in vivo* tumor inhibitory activities of the Maxatezo and Atezolizumab were examined in the treatment of MC38 mouse colorectal cancer model in C57BL/6J mice. The mice were inoculated with MC38 tumor cells and randomized grouping based on tumor volumes on day 7. Antibody control or drugs were administered with different doses. Tumors were collected, photographed and weighed at the end of the study on day 24.

**Table S1 Glycosylation profile of Maxatezo**

| **Clone ID** | **G0-GN (%)** | **G0F-GN (%)** | **G0 (%)** | **G0F (%)** | **Man-5 (%)** | **G1F (%)** | **G1F´ (%)** | **G2F (%)** | **Others (%)** |
| --- | --- | --- | --- | --- | --- | --- | --- | --- | --- |
| **C9** | 0.1 | 0.4 | 2.5 | 72.8 | 0.7 | 13.2 | 5.0 | 1.5 | 3.8 |
| **C10** | 0.2 | 0.5 | 2.6 | 70.6 | 0.8 | 14.0 | 5.3 | 1.6 | 4.4 |
| **C11** | 0.2 | 0.5 | 4.5 | 68.7 | 1.7 | 13.3 | 5.1 | 1.3 | 4.7 |
| **C12** | 0.1 | 0.5 | 2.4 | 73.8 | 0.7 | 12.6 | 4.8 | 1.3 | 3.8 |
| **C13** | 0.3 | 0.9 | 2.9 | 62.7 | 2.5 | 17.3 | 6.1 | 2.5 | 4.8 |
| **C14** | 0.1 | 0.5 | 2.6 | 71.9 | 0.7 | 13.5 | 5.1 | 1.5 | 4.1 |
| **C15** | 0.1 | 0.5 | 2.4 | 70.0 | 0.8 | 14.4 | 5.6 | 1.6 | 4.6 |
| **C16** | 0.2 | 0.5 | 2.6 | 71.7 | 0.7 | 13.5 | 5.1 | 1.5 | 4.2 |
| **C17** | 0.3 | 0.9 | 3.1 | 63.4 | 2.2 | 16.9 | 6.1 | 2.4 | 4.7 |
| **C18** | 0.2 | 0.5 | 2.6 | 70.0 | 0.7 | 14.6 | 5.6 | 1.7 | 4.1 |

Notes: C9 to C18 represent different stable cell lines. Abbreviations: G, galactose; F, fucose; Man, mannose; GN, N-acetylglucosamine. For example, G1F indicates Fc oligosaccharides in which the core heptasaccharide bears one terminal galactose and is also fucosylated.

**Table S2 Expression of antibodies by stable CHO cell pools**

| **Run No.** | **Culture Volume** | **Final Titer (g/L)** |
| --- | --- | --- |
| B01 Atezolizumab | 5L | 1.36 |
| B02 Atezolizumab | 2L | 1.30 |
| B03 Maxatezo | 2L | 4.39 |
| B04 Maxatezo | 2L | 4.07 |

Note: The stable cell pools expressing either Atezolizumab or Maxatezo were seeded in Bioreactors and the culture supernatants were harvested on Day 12. The antibodies in the culture supernatants were separated by analytic HPLC and the antibody concentrations were determined based on the areas of the peaks at OD 280.

**Table S3 Expression of antibodies by stable CHO cell lines**

| Clone ID | Initial VCD (×10^6^cells/mL) | pVCD (×10^6^cells/mL) | Final VCD (×10^6^cells/mL) | Final Viability  （%） | Peak Lactate (g/L) | Final Lactate(g/L) | Final Titer (g/L) | Culture Duration (days) |
| --- | --- | --- | --- | --- | --- | --- | --- | --- |
| C11 | 0.58 | 14.86 | 8.66 | 77.0 | 1.6 | 0.4 | 5.45 | 12 |
| C12 | 0.55 | 34.37 | 28.50 | 96.5 | 2.1 | 0.0 | 4.73 | 12 |
| C14 | 0.49 | 27.91 | 24.15 | 96.9 | 2.0 | 0.0 | 4.74 | 12 |
| C15 | 0.55 | 24.84 | 21.37 | 97.1 | 2.7 | 1.4 | 5.12 | 12 |

Note: The different clones were seeded in Bioreactors and the culture supernatants were harvested on Day 12. The antibodies in the culture supernatants were separated by analytic HPLC and the antibody concentrations were determined based on the areas of the peaks at OD 280.

**Table S4 HPLC-SEC Analysis**

| mAb |  | RT (min) | Area (units) | Height | % Area |
| --- | --- | --- | --- | --- | --- |
| Maxatezo | Monomer | 32.679 | 23431856 | 348923 | 99.470 |
|  | HMW | 26.959 | 124831 | 883 | 0.530 |
| Atezolizumab | Monomer | 32.915 | 21572737 | 315915 | 99.214 |
|  | HMW | 28.876 | 170858 | 898 | 0.786 |

**Table S5 Thermal stability evaluation of Atezolizumab and Maxatezo**

| **Sample** | | **T_m_1 (°C)** | | | **T_agg_** | | |
| --- | --- | --- | --- | --- | --- | --- | --- |
|  |  | **T_m_1 (°C)** | **Average T_m_1 (°C)** | **%CV T_m_1 (°C)** | **T_agg_ (°C)** | **Average T_agg_ (°C)** | **%CV T_agg_** |
| Maxatezo | Sample 1 | 70.89 | 71.01 | 0.16 | 70.89 | 71.20 | 0.44 |
|  | Sample 2 | 71.12 |  |  | 71.51 |  |  |
| Atezolizumab | Sample 3 | 63.50 | 63.55 | 0.08 | 60.75 | 60.70 | 0.1 |
|  | Sample 4 | 63.60 |  |  | 60.63 |  |  |
| **Sample** | | **T_m_2 (°C)** | | | **DLS** | | |
|  |  | **T_m_2 (°C)** | **Average T_m_2 (°C)** | **%CV T_m_2** | **Z-Ave. Dia. (nm)** | **PDI** | **Pk 1 Mode Dia. (nm)** |
| Maxatezo | Sample 1 | 86.15 | 86.18 | 0.03 | 8.42 | 0.071 | 8.96 |
|  | Sample 2 | 86.20 |  |  | 8.42 | 0.095 | 8.96 |
| Atezolizumab | Sample 3 | 87.40 | 87.65 | 0.29 | 8.90 | 0.182 | 8.29 |
|  | Sample 4 | 87.90 |  |  | 8.42 | 0.118 | 8.29 |

Note: T_m_ was monitored by Intrinsic Protein Fluorescence (IPF) and the onset T_agg_ was monitored by SLS at 473 nm. The size distribution of the samples was determined by DLS at 660 nm.

**Table S6 Experimental Design of *in vivo* Efficacy Study of MC38 Mouse Colorectal Cancer model**

| **Group** | **n** | **Treatment** | **Dose (mg/kg)** | **Dosing Route** | **Schedule** |
| --- | --- | --- | --- | --- | --- |
| 1 | 8 | Human IgG1 | 3.3 | i.p. | q3d × 6 |
| 2 | 8 | Maxatezo | 1 | i.p. | q3d × 6 |
| 3 | 8 | Maxatezo | 10 | i.p. | q3d × 6 |
| 4 | 8 | Atezolizumab | 10 | i.p. | q3d × 6 |

Note: n: animal number; i.p.: intraperitoneal injection; dosing volume: adjust dosing volume based on body weight at 10 μl/g; q3d dosing was administered once every 3 days; dosing was suspended when body weight loss reached 15% and resumed after body weight loss was less than 10%.

**Table S7 Anti-tumor Activity of PD-L1 Antibodies in the Treatment of MC38 Mouse Colorectal Cancer Model (Tumor Volume)**

| **Group** | **Animal**  **No.** | **Body Weight(g) ^a^** | | **Tumor Volume(mm^3^)^a^** | | **T/C (%)** | **TGI (%)** | ***P* ^b^** | ***P* ^c^** | **Complete tumor regression** |
| --- | --- | --- | --- | --- | --- | --- | --- | --- | --- | --- |
|  |  | **Before treatment (PG-D0)** | **After treatment (PG-D17)** | **Before treatment (PG-D0)** | **After treatment (PG-D17)** |  |  |  |  |  |
| Human IgG1 | 8 | 28.3±0.6 | 31.7±0.9 | 93±4 | 3912±348 | -- | -- | -- | -- | -- |
| Maxatezo  1mg/kg | 8 | 28.0±0.4 | 29.3±0.4 | 92±4 | 1068±433 | 26% | 74% | 0.000 | 0.018 | 1/8 |
| Maxatezo  10mg/kg | 8 | 28.2±0.5 | 28.4±0.5 | 92±5 | 97±83 | 2% | 98% | 0.000 | -- | 5/8 |
| Atezolizumab  10mg/kg | 8 | 28.4±0.4 | 30.4±1.0 | 93±4 | 1362±784 | 32% | 68% | 0.000 | 0.007 | 4/8 |

Note: a. Mean±SEM; b. compared with Human IgG1 control group; c. compared with Maxatezo 10mg/kg group. The differences between the mean values of tumor volumes for comparing groups are analyzed for significance using the mixed linear model and nonparametric (independent sample Kruskal-Wallis) test. P ≤ 0.05 was considered to be statistically significant.

**Table S8 Anti-tumor Activity of PD-L1 Antibodies in the Treatment of MC38 Mouse Colorectal Cancer Model (Tumor Weight)**

| **Groups** | **Animal No.** | **Tumor Weight (g)^a^** | **Tumor Weight Inhibition (%)** | ***P* ^b^** | ***P* ^c^** |
| --- | --- | --- | --- | --- | --- |
| Human IgG1 | 8 | 3.920 ± 0.381 | -- | -- | -- |
| Maxatezo  1mg/kg | 8 | 0.997 ± 0.425 | 75% | 0.176 | 1.000 |
| Maxatezo  10mg/kg | 8 | 0.098 ± 0.089 | 98% | 0.000 | -- |
| Atezolizumab  10mg/kg | 8 | 1.390 ± 0.816 | 65% | 0.039 | 1.000 |

Note: ^a^. Mean±SEM; ^b^ compared with Human IgG1 control group; ^c^ compared with Maxatezo 10mg/kg group. The differences between the mean values of tumor weights for comparing groups are analyzed for significance using the mixed linear model and nonparametric (independent sample Kruskal-Wallis) test. P ≤ 0.05 was considered to be statistically significant.
